# F89. COGNITIVE IMPAIRMENT WORSENING ACROSS AFFECTIVE TO PSYCHOSIS SPECTRUM: A COHORT STUDY OF UNIPOLAR/BIPOLAR DEPRESSION AND BIPOLAR SCHIZOAFFECTIVE DISORDERS

**DOI:** 10.1093/schbul/sby017.620

**Published:** 2018-04-01

**Authors:** Martin Roy, Marie-Pierre Strippoli, Caroline Vandeleur, Elsa Gilbert, Enrique Castelao, Jean-Michel Aubry, Pierre Marquet, Martin Preisig

**Affiliations:** 1 CERVO Brain Research Centre; 2 University Hospital of Lausanne; 3 Université Laval, CERVO Brain Research Centre; 4 University Hospital of Geneva

## Abstract

**Background:**

Bipolar (BPD), schizoaffective bipolar (SAM) and major depressive disorders (MDD) reveal large heterogeneity in terms of symptom expression, course and treatment response. This heterogeneity could be the source of a large variance of cognitive performance observed in these subjects. The aim of the present analyses was to compare the cognitive performance of patients with BPD, SAM, MDD and medical controls with adjustment for a comprehensive array of potential confounders. To go a step further we will simultaneously test the effects of multiple clinical characteristics including lifetime history and duration of psychotic symptoms, manic/hypomanic and depressive episodes, age of onset of disorder, current GAF score, time since remission of the last episode and presence of a depressive episode at the time of the assessment on the cognitive performance.

**Methods:**

Data stemmed from the Lausanne-Geneva Family and High-Risk study. Patients with BPD (n=62), SAM (n=22) and MDD (n=51) were interviewed every three years over a mean duration of follow-up of 12 years. All patients were assessed clinically with the semi-structured Diagnostic Interview for Genetic Studies (DIGS). The cognitive assessment was made with the MATRICS and the Victoria Stroop Test.

**Results:**

The global cognitive index (excluding Stroop result) shows that SAM subgroup had the lowest global score with 40.6 (SD=8.5), BPD 47.4 (SD=7.8) and MDD 49.7 (SD=8.7). A multiple linear regression accounting for several confounders such as comorbid psychiatric disorders and medication confirms that only SAM and BPD are statistically different from controls (p<0.001 and p<0.01 respectively). MDD did not differ from controls (p>0.05). Overall, patients with BPD or SAM but not with MDD showed poorer cognitive performance than controls in terms of the global score and speed of processing, verbal learning, working memory, visual learning, attention/vigilance and inhibition.

**Discussion:**

Our data confirm cognitive impairment in patients with BPD or SAM compared to controls after adjustment for a comprehensive array of potential confounder variables. We were able to evaluate the specificity of cognitive performance of psychotic, maniac and depressive dimensions of the major mood disorders within the same sample. Furthermore, these data stress that the presence of the “schizo dimension” concomitant to mania and depression contributes to worsening the cognitive performance in an additive manner.

